# Insulin resistance parameters in children born very preterm and adequate for gestational age

**DOI:** 10.1002/edm2.329

**Published:** 2022-02-23

**Authors:** Hernán García, Carolina Loureiro, Helena Poggi, Ivonne D'Apremont, Rosario Moore, José Tomás Ossa, María José Bruera, Soledad Peredo, Jacqueline Carvajal, Claudia Trincado, Alejandro Martínez‐Aguayo

**Affiliations:** ^1^ Pediatric Division School of Medicine Pontificia Universidad Católica de Chile Santiago Chile; ^2^ Complejo Asistencial Dr. Sótero del Río Puente Alto, Santiago Chile; ^3^ School of Medicine Pontificia Universidad Católica de Chile Santiago Chile

**Keywords:** HOMA, insulin resistance, metabolic syndrome, premature, PsiMS

## Abstract

**Background:**

Preterm neonates are at risk for metabolic syndrome later in life. Whether prematurity constitutes an independent risk factor for the development of cardiovascular disease and metabolic syndrome remains controversial.

**Objective:**

To compare anthropometric measures, cardiometabolic risk factors and insulin resistance variables between children who were born very preterm (VPT, <32 gestational weeks) and at term (Term, >37 gestational weeks) and adequate for gestational age (AGA).

**Methods:**

We designed a cross‐sectional cohort study, recruiting 120 children (5.0–8.5 years old) from the preterm clinic at Red de Salud UC‐Christus and Complejo Asistencial Dr. Sótero del Río, and term children from the community. We excluded children born small for gestational age, based on INTERGROWTH21. Anthropometrics data were classified using WHO reference standards. The homeostasis model assessment insulin resistance (HOMA‐IR) index, quantitative insulin sensitivity check index (QUICKI), triglyceride‐to‐HDL‐C ratio (TG/HDL‐C) and Pediatric Score Index for Metabolic Syndrome (PsiMS) were calculated.

**Results:**

VPT children born AGA had lower HDL cholesterol levels (*p *= .019) and a higher PsiMS score than those born at term (*p *= .043). We observed a higher percentage of children with HDL cholesterol ≤40 mg/dl (13.0% vs. 2.3%, *p *= .026) and BP ≥90th percentile among the VPT children than among the Term children (26.0% vs. 11.6%, *p *= .031).

**Conclusions:**

At school age, blood pressure was higher, and HDL‐C was lower among VPT children born AGA, suggesting a potential metabolic risk; therefore, it is essential to follow this group throughout their lives.

## Abstract 2

Prepubertal children born very preterm and adequate for gestational age had a lower proportion of individuals with HDL‐C higher than 40 mg/dl and a higher proportion of individuals BP >90th percentile than children born at term.

## 1 INTRODUCTION

Metabolic syndrome (MetS) is characterized by a combination of cardiometabolic risk factors, including central obesity, hyperglycaemia, hypertension and dyslipidaemia.^1^, ^2^, ^3^ It is associated with increased morbidity and all‐cause mortality due to diabetes mellitus and cardiovascular diseases.^2^, ^4^ Approximately 650 thousand people die annually in the USA from cardiovascular events, which are also the leading cause of death worldwide.^5^

Based on the Third National Health and Nutritional Survey, Ford et al.^1^ estimated that MetS affects 25% of US adults. However, the prevalence in children is more variable depending on the definition and population, making it more challenging to estimate. Loureiro et al.^6^ reported a MetS prevalence of 10.8% among schoolchildren in Santiago de Chile, which is consistent with other reported paediatric MetS prevalence rates.^7^ Among obese children, as expected, MetS is even more prevalent, at approximately 30%, and 75% of obese children meet at least one criterion for MetS.^7^ For this reason, it is essential to identify metabolic disorders at an early age to prevent long‐term damage.

There is growing evidence that foetal and early life events may result in permanent metabolic alterations,^8^, ^9^, ^10^, ^11^ and several studies have shown an association between low birth weight (LBW) and the development of MetS later in life.^4^, ^9^, ^11^, ^12^ It has also been reported that adults born prematurely show elevated arterial blood pressure (BP), altered glucose tolerance test results and lipid profiles, increased total body fat mass, and increased risk for cardiovascular diseases compared to individuals who were born after a full‐term pregnancy. Nevertheless, most of these studies included preterm subjects who were small for gestational age (SGA) and/or LBW without distinction. Thus, it remains controversial whether prematurity constitutes an independent risk factor for the development of cardiovascular disease and MetS. This study aimed to compare anthropometric measures and metabolic syndrome variables between very preterm and term children who were born adequate for gestational age.

## 2 METHODS

### 2.1 Study design and participants

This cross‐sectional study included prepubertal children aged 5.0–8.5 years from different urban areas in Santiago de Chile. Preterm children were recruited at the preterm clinic at Red de Salud UC‐Christus and Complejo Asistencial Dr. Sótero del Río, and term children were invited from the community. The two groups had similar socioeconomic backgrounds.

Data from the newborn period were obtained from neonatal/infant control cards. Gestational age was assessed according to the first day of the last menstrual period and early prenatal ultrasonography results. Very preterm (VPT) birth was defined as birth at a gestational age <32 weeks, and term birth was defined as birth at 37 or more weeks of gestation. The children were considered born adequate for gestational age (AGA) when their birth weight was more than −2.0 standard deviation scores (SDSs) as calculated with INTERGROWTH21.^13^

The exclusion criteria were the presence of at least one of the following conditions in the children: renal disease, diabetes mellitus, hepatic failure and calcium disorders as established by the measurement of serum creatinine, calcium, alkaline phosphatase, bilirubin, alanine aminotransferase, aspartate aminotransferase and albumin, thyroid‐stimulating hormone, insulin growth factor 1, and parathyroid hormone.

### 2.2 Anthropometrics

All recruited subjects underwent a complete physical examination and were evaluated by a paediatric endocrinologist, nephrologist, and cardiologist at the Pontificia Universidad Católica de Chile from January 2016 to August 2018. Height was measured using a stadiometer (Health o metre model 402 KL) with 0.1‐cm precision, and weight was measured using a precision scale (Omron model HBF‐510). Height and body mass index (BMI) are expressed according to World Health Organization (WHO) references. Pubertal stage, as assessed by medical examination, was classified according to Tanner, considering girls with Tanner 1 breast development and boys with Tanner 1 gonadal development (testes <4 cc) to be prepubertal. Abdominal circumference (AC) was measured using standardized procedures as recommended by the WHO (midpoint between the lower costal margin and iliac crest).

### 2.3 Biochemical parameters

The following parameters were measured in serum obtained after 8–12 h of fasting: insulin by an electrochemiluminescence assay (Cobas, Roche Diagnostics GmbH), glucose by an enzymatic assay (Roche, Hitachi) and total cholesterol, high‐density lipoprotein cholesterol (HDL‐C), and triglycerides (TGs) by colorimetric assays (Roche, Hitachi). We calculated the homeostasis model assessment insulin resistance (HOMA‐IR) index, the quantitative insulin sensitivity check index (QUICKI),^14^ and the TG‐to‐HDL‐C ratio (TG/HDL‐C). HOMA‐IR was obtained by multiplying fasting insulin and glucose, divided by 22.5, and the QUICKI was calculated using the formula 1/(log fasting insulin [μU/ml] +log fasting glucose [mg/dl]).^15^

### 2.4 Nutritional status and cardiovascular risk

Based on WHO recommendations, nutritional status indicators in children aged between 5 and 19 years were classified based on the body mass index (BMI) SDS (Standard Deviation Score): overweight when BMI‐for‐age >1 SDS and obesity >2 SDS above the WHO Growth Reference median.

The following antecedents were considered to be cardiovascular disease risk factors: family history (first or second degree relatives either of the mother or the father) of dyslipidaemia, coronary artery disease earlier than 55 years old, cerebrovascular diseases and/or type 2 diabetes. In addition, unhealthy lifestyle environment, such as intradomiciliary tobacco habits, prolonged screen time, and poor physical activity, were recorded. Data were obtained from the mother or father through a questionnaire during physical examination.

### 2.5 Definition of metabolic syndrome

MetS was defined, according to Cook's criteria, as when three or more of the following conditions were present: abdominal circumference ≥90th percentile, BP ≥90th percentile, fasting glycaemia ≥100 mg/dl, HDL cholesterol ≤40 mg/dl and triglycerides ≥110 mg/dl.^16^

BP percentiles were calculated and categorized according to the American Academy of Pediatrics 2017^17^ criteria as normal (50th percentile), elevated (>90th percentile), stage 1 hypertension (HTN) (≥95th percentile) and stage 2 HTN (≥95th percentile +12 mmHg).

The Pediatric Score Index for Metabolic Syndrome (PsiMS) score,^18^ which is a practical and accurate score for evaluating MetS among obese youth, was calculated using the following formula: (2xWaist/Height) + (Glucose (mmol/l)/5.6) + (Triglycerides (mmol/l)/1.7) + (Systolic BP/130) − (HDL‐C (mmol/l)/1.02).

### 2.6 Carotid intima‐media thickness

Carotid intima‐media thickness (cIMT) was measured by a trained nurse and a physician using a General Electric Vivid E9 Echocardiography machine and a linear probe with a frequency range of 6–15 MHz. MUST software with the Auto IMT program was used. Measurements of the posterior walls of both common carotid arteries were obtained 10 mm proximal to the carotid bulb. In the longitudinal view, at least 200 measurement points were selected for at least three different insonation angles (i.e. approximately 150, 105 and 60 degrees for the right common carotid artery and 210, 255 and 300 degrees for the left common carotid artery).

### 2.7 Statistics

SPSS and Prism 8.2 were used for statistical analysis. The results are presented as the median with interquartile range and as the mean with standard deviations. To evaluate statistically significant differences between groups, *p* values from the Mann–Whitney *U* test or independent samples *t*‐test were obtained.

We calculated the Pearson correlation coefficient (*r*) to analyse the associations between BMI (SDS) and metabolic variables, including abdominal circumference (SDS), glycaemia (mg/dl), HDL‐C (mg/dl), TG (mg/dl), SBP (percentile), DBP (percentile), TG/HDL‐C ratio, HOMA, the QUICKI and the PsiMS for the ‘Very Preterm’ and ‘Term’ groups. After controlling for abdominal circumferences (SDS), a partial correlation was also performed.

### 2.8 Ethics

The study was approved by the Ethics Committee of the Faculty of Medicine, Pontificia Universidad Católica de Chile (Project ID: 16‐050) according to the Helsinki Declaration. The parents or legal representatives of the children signed informed consent forms before the children entered the study.

## 3 RESULTS

### 3.1 General characteristics

Among the 120 children included, 77 (64.2%) were VPT (gestational age, 29 ± two weeks), and 43 (35.8%) were born at term (gestational age, 39 ± one week). The groups were comparable in terms of chronological age, height SDS, abdominal circumference, BMI percentile and BW‐SDS, as described in Table 1.

**TABLE 1:** General characteristics of the study population

	Very Preterm (*n* = 77)		Term (*n* = 43)		*p* value
	Median [IQ]	Mean (SD)	Median [IQ]	Mean (SD)	
Neonatal data					
Gestational age (weeks)	29 [28 to 30]	28.9 (2.1)	39 [30 to 40]	38.9 (1.0)	.001
Birth weight (SDS)	0.17 [−0.51 to 0.82]	0.12 (0.94)	0.42 [−0.06 to 1.02]	0.39 (0.71)	.121
Birth length (SDS)	−0.16 [−0.75 to 0.33]	−0.28 (1.12)	0.62 [−0.32 to 1.26]	0.43 (1.11)	.001
Anthropometric data					
Age (years)	6.5 [5.6 to 7.2]	6.5 (0.9)	6.5 [5.8 to 7.8]	6.7 (1.1)	.299
Height (SDS)	−0.2 [−0.68 to 0.47]	−0.16 (0.79)	0.09 [−0.52 to 0.79]	0.11 (1.0)	.115^a^
BMI (SDS)	0.63 [−0.24 to 1.35]	0.46 (1.16)	0.7 [−0.29 to 1.28]	0.62 (1.1)	.440^a^
Abdominal circumference (cm)	56.0 [54.0 to 61.0]	58.5 (7.1)	56.5 [53.5 to 63.0]	59.1 (7.9)	.710
Abdominal circumference (percentile)	43.3 [24.5 to 67]	44.4 (25.0)	48.4 [31.9 to 80]	53.3 (29.2)	.086
Abdominal circumference/height	0.49 [0.46 to 0.53]	0.49 (0.05)	0.48 [0.46 to 0.51]	0.49 (0.06)	.399
Cardiovascular data					
SBP (mmHg)	100 [95 to 106]	101 (7)	99 [93 to 105]	99 (7)	.353^a^
SBP (percentile)	72 [54 to 85]	68.8 (22.1)	67 [48 to 79]	64.1 (20.9)	.185
DBP (mmHg)	59 [54 to 63]	58.7 (6.9)	60 [56 to 62]	58.8 (4.9)	.854
DBP (percentile)	60 [46 to 78]	60.6 (20.8)	58 [49 to 70]	58.8 (15.9)	.666^a^
Heart rate per min	94 [85 to 106]	94.3 (12.6)	91 [84 to 98]	91.0 (11.7)	.156^a^
cIMT (mm)	0.44 [0.42 to 0.46]	0.44 (0.34)	0.44 [0.42 to 0.48]	0.44 (0.05)	.801
Abdominal aorta velocity (m/s)	1.02 [0.91 to 1.14]	1.05 (0.26)	1.00 [0.82 to 1.15]	0.98 (0.19)	.413

Note

BMI, body mass index; cIMT, carotid intima‐media thickness; DBP, diastolic blood pressure; SBP, systolic blood pressure; SDS, standard deviation score.

^a^ *p* values are from the Mann–Whitney *U* test or independent samples *t*‐test.

### 3.2 Nutritional status and family history of cardiovascular disease

The nutritional status distribution was similar in the two groups (Pearson's chi‐squared test, *p *= .810). For VPT versus T children, severe thinness was found in 1 subject (1.3%) versus none, thinness in 7 versus 3 (9.1% and 7.0% respectively), normal weight in 46 versus 25 (59.7% and 58.1% respectively), overweight in 18 versus 10 (23.4% and 23.3% respectively) and obesity in 5 versus 5 (6.5% and 11.6% respectively).

Family history of CVD showed a similar distribution among VPT and T children: hypertension: 76% versus 65.1%, *p *= .100; familial dyslipidaemia: 34.7% versus 44.2%, *p *= .152; coronary artery disease earlier than 55 years old: 11.8% versus 4.9%, *p *= .106; cerebrovascular diseases: 11.8% versus 23.3%, *p *= .05; diabetes type 2: 56.6% versus 51.2%, *p *= .284; and intradomiciliary tobacco habits; 57.9% versus 53.5%, *p *= .319.

### 3.3 Physical activity and screen time

The median number of hours of physical activity per day was 4.0 h [3.0–6.0 h] in the VPT group and 5.0 h [3.5–5.5 h] in the T group (*p *= .157), and that of screen time per day was 2.0 h [1.0–3.0 h] in the VPT group and 2.0 h [1.5–4.0 h] in the T group (*p *= .095).

### 3.4 Clinical blood pressures, cIMT and abdominal aorta velocity

As shown in Table 1, systolic (*p *= .353) and diastolic (*p *= .854) BP, cIMT (*p *= .801) and abdominal aorta velocity (*p *= .413) were similar in the two groups. The BP category distribution of the VPT and T groups showed analogous results (Pearson's chi‐squared test, *p *= .169): 57 VPT children with normal BP versus 38 T children (74.0% and 88.3%), 10 VPT children with elevated BP versus 3 T children (13.0% and 7.0%), and 10 VPT children with stage 1 HTN versus 2 T children (13.0% and 4.7%).

### 3.5 Insulin resistance indexes and metabolic syndrome score

We did not find differences in fasting glycaemia (*p *= .928), insulinaemia (*p *= .907), TG/HDL‐cholesterol ratio (*p *= .116), HOMA‐IR (*p *= .945) or QUICKI (*p *= .921) between the two groups. However, VPT children had lower HDL cholesterol levels (*p *= .019) and a higher PsiMS score than those born at term (*p *= .043), as shown in Table 2.

**TABLE 2:** Insulin resistance parameters in children who were born very preterm and at term

	Very Preterm (*n* = 77)		Term (*n* = 43)		*p* value
	Median [IQ]	Mean (SD)	Median [IQ]	Mean (SD)	
Lipid profile					
Total cholesterol (mg/dl)	148 [132–165]	150 (24.8)	157 [136–171]	156.9 (26.1)	.216^a^
TG (mg/dl)	61[46–79]	66.8 (30.8)	57 [44–72]	61.7 (25.9)	.356
HDL‐C (mg/dl)	54 [46–62]	53.7 (11.0)	58 [50–66]	58.7 (10.6)	.019^a^
LDL‐C (mg/dl)	82 [69–97]	83.7 (22.6)	83 [71–94]	85.8 (22.2)	.825
VLDL‐C (mg/dl)	12 [9−16]	13.3 (6.2)	11 [9–14]	12.3 (5.1)	.407
Insulin resistance indexes and metabolic syndrome score					
Glycaemia (mg/dl)	84 [80–88]	84 (6.8)	84 [80–88]	83 (7.4)	.928
Insulin (μU/ml)	4.7 [3.4–7.0]	5.9 (3.7)	5.1 [3.6–7.1]	5.6 (2.9)	.907
TG/HDL ratio	1.06 [0.83–1.55]	1.36 (0.9)	0.88 [0.75–1.35]	1.09 (0.49)	.116
HOMA	1.02 [0.68–1.56]	1.26 (0.82)	1.06 [0.7–1.55]	1.18 (0.64)	.945
QUICKI	0.17 [0.16–0.18]	0.17 (0.02)	0.17 [0.16–0.18]	0.17 (0.04)	.921
PsiMS	1.66 [1.38–1.87]	1.69 (0.49)	1.48 [1.24–1.73]	1.50 (0.39)	.043
Other biochemical variables					
GOT (IU/L)	29[25–32]	29.5 (5.4)	28[25–30]	30.0 (11.5)	.246
GPT (IU/L)	16[12–19]	16.6 (6.2)	15[12–18]	18.3 (15.1)	.530
Microalbumin/Cr (mg/g)	8.6[5.4–23]	19.2 (29.8)	7.2[5.5–13.3]	25.0 (71.5)	.425

Note

Insulin (μU/ml) * 6.945 = pmol/L, glycaemia (mg/dl) * 0.0555 = mmol/L. For total, HDL, and LDL cholesterol (mg/dl) * 0.0259 = mmol/L; and for TG * 0.0113.

Abbreviation: HDL‐C, high‐density lipoprotein cholesterol; PsiMS, Pediatric siMS score; TG, triglyceride.

^a^ *p* value is from Mann–Whitney *U* test or independent samples *t*‐test.

We observed a higher percentage of children with HDL cholesterol ≤40 mg/dl (13.0% vs. 2.3%, *p *= .026) and BP ≥90th percentile in VPT children than in T children (26.0% vs. 11.6%, *p *= .031). However, the distribution of MetS components were not significantly different between the two groups (Table 3).

**TABLE 3:** Prevalence of the risk factors that determine metabolic syndrome (MetS) in children born very preterm and at term

	Very preterm (*n* = 77)	Term (*n* = 43)	*p* value
AC ≥90th percentile	3.9%	11.6%	.052
Glycaemia ≥100 mg/dl	0.0%	0.0%	NC
HDL‐C ≤ 40 mg/dl	13.0%	2.3%	.026
TG ≥110 mg/dl	10.4%	4.7%	.140
BP ≥90th percentile	26.0%	11.6%	.031
N° MetS components			
0	63.6%	76.7%	.730
1	23.4%	16.3%	
2	9.1%	7.0%	
3	3.9%	0.0%	

Note

*p* values were calculated using two‐sample normal proportion tests (one‐tailed).

Abbreviations: AC, abdominal circumference; BP, blood pressure (systolic and/or diastolic); HDL‐C, high‐density lipoprotein cholesterol; TG, triglyceride; NC, not calculated.

Pearson correlations between BMI (SDS) and metabolic variables are presented by group (Table 4). In the Very Preterm group, BMI (SDS) was associated with a larger number of metabolic variables than in the Term group (TG, TG/HDL ratio and QUICKI), and this association persisted after controlling for abdominal circumference (SDS).

**TABLE 4:** Pearson association (*r*) between BMI (SDS) and metabolic variables for the ‘Very Preterm’ and ‘Term’ groups

	Very Preterm (*n *= 77)		Term (*n* = 43)	
	BMI (SDS)		BMI (SDS)	
	Unaj.	Adj.^a^	Unaj.	Adj.^a^
Abdominal circumference (SDS)	.23*	–	.52**	
Glycaemia (mg/dl)	NS	NS	NS	NS
HDL‐C (mg/dl)	NS	NS	NS	NS
TG (mg/dl)	.33**	.36**	NS	NS
SBP (percentile)	.24*	.29*	.34*	NS
DBP (percentile)	NS	NS	NS	NS
TG/HDL ratio	.37**	.39**	NS	NS
HOMA‐IR	.44***	.42***	.45**	.36*
QUICKI	−.42***	−.39**	NS	NS
PsiMS	.45***	.46***	.53**	.50**

Note

*p* values *<.05; **<.01; ***<.001

Abbreviations: BP, blood pressure (systolic and/or diastolic); HDL‐C, high‐density lipoprotein cholesterol; HOMA‐IR, homeostatic model assessment; NS, not significant; PsiMS, Pediatric Simple Metabolic Syndrome score; QUICKI, Quantitative Insulin Sensitivity Check Index; TG, triglyceride.

^a^ After controlling for abdominal circumference (SDS).

## 4 DISCUSSION

### 4.1 What is known?

The association between MetS parameters and prematurity has been demonstrated previously for preterm SGA infants and for groups that included a mixed population of AGA and SGA individuals.^19^, ^20^, ^21^, ^22^ However, Markopoulou et al.^8^ suggest that the evidence is less strong regarding preterm birth and its link with the development of components of MetS.

### 4.2 What is new about this work?

Prepubertal children born VPT and AGA had a lower proportion of individuals with HDL‐C higher than 40 mg/dl (mmol/L) and a higher proportion of individuals BP >90th percentile than children born at term. This finding suggests that in VPT‐AGE children, at least two characteristics associated with MetS can be identified early in life.

The two groups were homogeneous in terms of anthropometric characteristics, family history of CVD, family history of type 2 diabetes, intradomiciliary tobacco use, physical activity, and screen time. The median clinical BP, cIMT and abdominal aorta velocity were similar between the two groups. In agreement with our findings, Mohlkert et al.^23^ reported that in 6‐year‐old children born extremely preterm, no signs of accelerated intima‐media thickening or arterial stiffening were found. However, the same author reported years later that children born extremely preterm exhibit higher estimated pulmonary vascular resistance and altered right heart structure and function compared with children born at term.^24^ Other authors reported that at eleven years old, patients had increased cIMT and systolic BP. It is unknown whether these changes are due to preterm birth and rapid maturation of the skin or to nutritional factors.^25^ Recently, a meta‐analysis described several risk factors in the first 1000 days of life associated with increased cIMT during childhood: SGA had the most consistent relationship with increased cIMT.^26^ Being born SGA was independently associated with increased aortic IMT after controlling for perinatal, anthropometric and biochemical determinants in linear regression models.^27^ As both SGA and AGA children born preterm could develop an unfavourable and altered cardiovascular risk profile, implementation of routine cardiovascular follow‐up programmes might be warranted.

No differences were observed in the HOMA‐IR index, QUICKI or TG/HDL‐C ratio between VPT and Term children at this age. However, the VPT group had a lower HDL‐C concentration and a higher proportion of individuals with BP ≥90th percentile than the Term group.

We did not find significant differences in the diagnosis of MetS (according to the Cook criteria definition), insulin resistance parameters or cIMT between prepubertal children born VPT and AGA and those born at term with similar characteristics. Nevertheless, the main finding of this study is that already at this prepubertal age, there are some parameters of MetS that are altered: the proportion of children with low HDL‐C and elevated BP was higher in the VPT group, suggesting a potential cardiometabolic risk in the future; therefore, it is essential to follow this group throughout their lives.

Central obesity is one of the most well‐recognized components of MetS, and it has been closely associated with LBW.^28^ In our cohort, a similar distribution of obesity and overweight was observed in the two groups. We want to highlight that even though the two groups had similar BMIs, a stronger correlation with MetS parameters was observed for infants born VPT, even after controlling for the results for abdominal circumference. However, accelerated weight gain during infancy among preterm children may be a critical contributor to obesity later in life.^29^

The most frequent MetS parameters identified in this cohort were elevated BP and lower HDL‐C concentrations. The association between HTN in childhood and prematurity has been previously described.^30^ In a study by Heidemann et al.,^9^ BP was found to be altered in 57.5% of the participants, and elevated BP was the most prevalent MetS parameter in adults born preterm. This study showed that the mean value of HDL‐C, a protective parameter against MetS, was significantly lower in the VPT group than in the T group. As shown in other studies, a lower concentration of HDL‐C is characteristic of insulin resistance, and it is also related to increased cardiovascular risk in adulthood.^31^, ^32^, ^33^

In addition to this finding, the PsiMS score was significantly different between the VPT and Term groups. The PsiMS is a modified continuous score based on the original siMS score described for adults and is used for the evaluation of MetS in the paediatric population. It is easy to use in clinical practice and has a high correlation with more complex scores.^16^ The PsiMS includes HDL‐C in its formula, which could explain the significance of this result.

A recent systematic review and meta‐analysis of 43 studies (18,295 adults born preterm and 294,295 born at term) by Markopoulou et al.^8^ showed similar results regarding arterial BP; however, they also described an association between preterm birth and elevated HOMA‐IR values, increased levels of fasting glucose, insulin and total cholesterol levels, a higher percentage of fat mass and higher IMC. They concluded that preterm birth is strongly associated with many components of MetS and cardiovascular disease in adult life, but they did not differentiate the results for those born AGA and SGA. Balasuriya et al.,^4^ in a cohort study of 189 young people from 25 to 28 years old, looked for metabolic outcomes in adults born preterm with very LBW (<1500 g) or SGA at term. They demonstrated that VPT individuals had higher insulin, HOMA‐IR, systolic and diastolic BP and lower HDL‐C. Sullivan et al.^34^ demonstrated that in young adults born AGA, individuals born VPT had early cardiovascular risk factors, such as elevated BP, but did not meet the criteria for MetS. Similarly, a cohort study conducted in all 4,193,096 singletons born in Sweden between 1973 and 2014 unequivocally showed that gestational age at birth was inversely associated with both type 1 and type 2 diabetes risk. The adjusted hazard ratios (HRs) for type 1 and type 2 diabetes at age >18 years associated with preterm birth compared with full‐term birth were 1.21 and 1.26, respectively, and those at age 18–43 years were 1.24 and 1.49 respectively. This study did not differentiate between being born AGA and SGA.^19^

### 4.3 Study limitations

One limitation could be the relatively small number of preterm subjects included in our cohort. It could impact our ability to detect statistically significant associations for some of the parameters measured. However, it is challenging to find a large group of VPT and AGA children and follow them throughout their lives, which is a remarkable feature of our study. In addition, we included only prepubertal children, that is, children who have not reached puberty. Efforts should be made to replicate this study, including more children and following them during adolescence and adulthood.

## 5 CONCLUSION

In summary, our study showed that children born VPT and AGA may have some MetS indicators at an early prepubertal age. Suggesting that they could have future cardiometabolic risk; therefore, they require clinical follow‐up throughout their lives to prevent the development of adverse pathologies and promote healthy lifestyles. More studies are needed to evaluate indicators of insulin resistance at an older age to identify more associations between preterm birth and MetS.

## CONFLICT OF INTEREST

The authors have no financial or nonfinancial conflicts of interest to declare in association with this work. The authors declare no conflicts of interest.

## AUTHOR CONTRIBUTION

**Hernán García:** Conceptualization (lead); Formal analysis (equal); Writing – review & editing (supporting). **Carolina Loureiro:** Conceptualization (equal); Writing – original draft (equal); Writing – review & editing (equal). **Helena Poggi:** Conceptualization (supporting); Project administration (lead); Supervision (lead); Writing – original draft (equal); Writing – review & editing (lead). **Ivonne D’Apremont:** Data curation (lead); Investigation (equal); Resources (equal); Validation (equal); Writing – review & editing (equal). **Rosario Moore:** Data curation (lead); Formal analysis (supporting); Validation (equal); Writing – original draft (equal); Writing – review & editing (supporting). **José Tomás Ossa:** Formal analysis (supporting); Project administration (supporting); Software (supporting); Validation (equal); Visualization (equal). **María José Bruera:** Formal analysis (equal); Software (supporting); Validation (supporting); Visualization (supporting); Writing – original draft (equal). **Soledad Peredo:** Conceptualization (equal); Data curation (equal); Formal analysis (equal); Software (equal); Writing – original draft (equal). **Jacqueline Carvajal:** Methodology (equal); Resources (equal); Software (lead); Validation (equal). **Claudia Trincado:** Conceptualization (equal); Data curation (equal); Formal analysis (equal); Software (equal). **Alejandro Martinez‐Aguayo:** Conceptualization (lead); Data curation (lead); Formal analysis (lead); Funding acquisition (lead); Investigation (equal); Methodology (equal); Project administration (equal); Resources (lead); Supervision (lead); Validation (lead); Writing – original draft (lead); Writing – review & editing (lead).

## ETHICS STATEMENT

The study was approved by the Ethics Committee of the Faculty of Medicine, Pontificia Universidad Católica de Chile (Project ID: 16–050) according to the Helsinki Declaration. The parents or legal representatives of the children signed informed consent forms before the children entered the study.

## Data Availability

While the data are not available in a public repository, other researchers can access them through Alejandro Martinez‐Aguayo – alemarti@med.puc.cl.
